# Relationships between educational attainment, hypertension, and amyloid negative subcortical vascular dementia: The brain-battering hypothesis

**DOI:** 10.3389/fnins.2022.934149

**Published:** 2022-08-05

**Authors:** Song Hwangbo, Young Ju Kim, Yu Hyun Park, Hee Jin Kim, Duk L. Na, Hyemin Jang, Sang Won Seo

**Affiliations:** ^1^Department of Neurology, Samsung Medical Center, Sungkyunkwan University School of Medicine, Seoul, South Korea; ^2^Neuroscience Center, Samsung Medical Center, Seoul, South Korea; ^3^Department of Health Sciences and Technology, Samsung Advanced Institute for Health Sciences & Technology (SAIHST), Sungkyunkwan University, Seoul, South Korea; ^4^Stem Cell and Regenerative Medicine Institute, Samsung Medical Center, Seoul, South Korea; ^5^Samsung Alzheimer Convergence Research Center, Samsung Medical Center, Seoul, South Korea; ^6^Department of Intelligent Precision Healthcare Convergence, Sungkyunkwan University School of Medicine, Suwon, South Korea

**Keywords:** Alzheimer's disease, vascular dementia, education, hypertension, vascular risk factor

## Abstract

**Purpose:**

Many epidemiological studies suggest that lower education levels and vascular risk factors increase the likelihood of developing Alzheimer's disease dementia (ADD) and subcortical vascular dementia (SVaD). However, whether the brain-battering hypothesis can explain the relationship between education levels and the clinical diagnosis of dementia remains controversial. The objective of this study was to investigate whether vascular risk factors mediate the association between education level and the diagnosis of amyloid-beta positive (Aβ+) ADD and amyloid-beta negative (Aβ-) SVaD.

**Methods:**

We analyzed 376 participants with Aβ normal cognition (Aβ- NC), 481 with Aβ+ ADD, and 102 with Aβ- SVaD. To investigate the association of education level and vascular risk factors with these diagnoses, multivariable logistic regression analysis was used, with age, sex, and *APOE* ε4 carrier status used as covariates. Path analysis was performed to investigate the mediation effects of hypertension on the diagnosis of Aβ- SVaD.

**Results:**

The Aβ- SVaD group (7.9 ± 5.1 years) had lower education levels than did the Aβ- NC (11.8 ± 4.8 years) and Aβ+ ADD (11.2 ± 4.9 years) groups. The frequencies of hypertension and diabetes mellitus were higher in the Aβ- SVaD group (78.4 and 32.4%, respectively) than in the Aβ- NC (44.4 and 20.8%) and Aβ+ ADD (41.8 and 15.8%, respectively) groups. Increased education level was associated with a lower risk of Aβ- SVaD [odds ratio (OR) 0.866, 95% confidence interval (CI), 0.824–0.911], but not Aβ+ ADD (OR 0.971, 95% CI 0.940–1.003). The frequency of hypertension was associated with a higher risk of developing Aβ- SVaD (OR 3.373, 95% CI, 1.908–5.961), but not Aβ+ ADD (OR 0.884, 95% CI, 0.653–1.196). In the path analysis, the presence of hypertension partially mediated the association between education level and the diagnosis of Aβ- SVaD.

**Conclusion:**

Our findings revealed that education level might influence the development of Aβ- SVaD through the brain-battering hypothesis. Furthermore, our findings suggest that suitable strategies, such as educational attainment and prevention of hypertension, are needed for the prevention of Aβ- SVaD.

## Introduction

Alzheimer's disease dementia (ADD), characterized by amyloid-beta (Aβ), and subcortical vascular dementia (SVaD), indicated by extensive white matter hyperintensities (WMH), are the two most common causes of dementia. Low educational attainment has been regarded as a risk factor for these two types of dementia (Katzman, 1993; Caamaño-Isorna et al., 2006; Sharp and Gatz, 2011). As one of the supporting hypotheses, the brain-battering hypothesis was suggested (Del Ser et al., 1999). This theory postulates that individuals with lower education are likely to have lower socioeconomic status (SES) that is subsequently related to lower health consciousness, bad habits, exposure to various toxins, and uncontrolled vascular risk factors than those with higher education and associated higher SES. Thus, individuals with lower education and more vascular risk factors are likely to develop brain pathologic burdens, which result in the development of dementia (Del Ser et al., 1999; Meng and D'Arcy, 2012). This brain-battering hypothesis is in comparison with the cognitive reserve (CR) theory which explains that lower educated individuals cannot cope with pathological changes in the brain and consequently are more likely to develop dementia. Thus, we consider that the CR theory explains the difference in clinical manifestation according to education when pathologic burdens are equal, while the brain-battering hypothesis may explain the difference in the probability of pathologic burden development according to education levels. Compared to the CR theory, the brain-battering hypothesis has been scarcely studied, especially in terms of its relation to two major pathologies of dementia.

To understand how a brain-battering hypothesis works in each phenotype, it is necessary to entangle the association between education, related risk factors, and each type of dementia. In fact, a previous study investigated this relationship, demonstrating that the association between education and risk of dementia was independent of SES, vascular or lifestyle characteristics (Ngandu et al., 2007). However, this study did not discriminate against dementia subtypes, which limits a separate interpretation in ADD and SVaD.

Considering that the brain-battering hypothesis is closely related to vascular risk factors, in this study, we aimed to examine the brain-battering hypothesis in two dementia subtypes by investigating whether two well-known vascular risk factors—hypertension and diabetes mellitus (DM)—mediate the association between education levels and the development of ADD and SVaD. Here, we used Aβ positron emission tomography (PET) imaging to discriminate a pure form of ADD (Aβ+ ADD) and SVaD (Aβ- SVaD) from patients with mixed or other possible pathologies. Many studies have suggested that vascular risk factors increase the development of dementia, especially SVaD (Launer et al., 2000; Kivipelto et al., 2005; Duron and Hanon, 2008; Rönnemaa et al., 2011; Caruso et al., 2019; Moretti and Caruso, 2020; Malik et al., 2021). However, it is controversial that vascular risk factors are directly associated with AD pathology (Chui et al., 2011). Therefore, we hypothesized that the brain-battering hypothesis would explain the relationship between education levels and the development of dementia in patients with Aβ- SVaD, rather than in patients with Aβ+ ADD (Scarmeas et al., 2003).

## Materials and methods

### Participants

We consecutively recruited 2,274 participants from the memory clinic in the Department of Neurology at the Samsung Medical Center (SMC) in Seoul, Korea, between 2008 and 2020. All participants underwent brain magnetic resonance imaging (MRI) and Aβ PET, including ^11^C-PiB PET, ^18^F-florbetaben PET, and ^18^F-flutemetamol PET. Among them, 464 were normal cognition [NC], 589 were ADD, and 177 were SVaD. Participants with ADD met the National Institute on Aging-Alzheimer's Association diagnostic criteria (McKhann et al., 2011). Participants with SVaD met the criteria described in the Diagnostic and Statistical Manual of Mental Disorders–Fourth Edition (Bell, 1994) and had severe WMHs on fluid-attenuated inversion recovery (FLAIR) images (Kalaria and Erkinjuntti, 2006), which satisfied the following criteria: (1) WMH ≥10 mm in the periventricular white matter (caps or rim); and (2) WMH ≥25 mm (maximum diameter) in the deep white matter, consistent with extensive white matter lesions or diffusely confluent lesions. We excluded participants who met any of the following conditions: (1) WMH due to etiologies other than vascular pathology, such as radiation injury, multiple sclerosis, leukodystrophy, or metabolic/toxic disorders; and (2) the presence of cerebral infarction, including large territory infarction and small cortical infarction, brain tumor, or vascular malformation on MRI. Participants with NC comprised healthy controls who visited the memory clinic for early prevention of dementia, healthy volunteers for comprehensive dementia evaluation, and participants with subjective cognitive complaints. All participants with NC met the following criteria: (1) no medical history that is likely to affect cognitive function based on Christensen's health screening criteria (Christensen et al., 1991), (2) no objective cognitive impairment from a comprehensive neuropsychological test battery on any cognitive domains (at least –1.0 SD above age-adjusted norms on any cognitive tests); (3) independence in activities of daily living; and (4) no structural lesions or severe WMH on brain MRI.

All participants were assessed through clinical interviews and neurological examinations, and clinical diagnoses were established by consensus among a multidisciplinary team. Blood tests included complete blood count, blood chemistry, vitamin B12/folate measurement, syphilis serology, thyroid function test, and *APOE* genotyping. Participants were excluded if they had territorial infarctions, cortical strokes, brain tumors, or vascular malformations on MRI.

### Standard protocol approvals, registrations, and participant consent

Written informed consent was obtained from all participants. This study was approved by the Institutional Review Board of the SMC. All procedures were performed in accordance with the approved guidelines.

### Acquisition of amyloid PET and data analysis

All participants underwent Aβ PET: ^11^C-PiB PET scans were conducted in 117 participants, ^18^F-florbetaben PET scans in 521 participants, and ^18^F-flutemetamol PET scans in 592 participants at the SMC using a Discovery STE PET/computed tomography scanner (GE Medical Systems, Milwaukee, WI, United States). For ^11^C-PiB PET, a 30-min static emission PET scan was performed 60 min after a bolus injection of a mean dose of 420 MBq. For ^18^F-florbetaben PET and ^18^F-flutemetamol PET, a 20-min emission PET scan in dynamic mode (consisting of 4 × 5 min frames) was performed 90 min after an injection of a mean dose of 311.5 MBq and 197.7 MBq for ^18^F-florbetaben and ^18^F-flutemetamol, respectively.

^11^C-PiB PET was regarded as positive if the global PiB uptake value was >1.5 (Lee et al., 2011). Florbetaben PET was classified as positive if the amyloid-plaque load on the florbetaben PET scan was visually rated as 2 or 3 on the brain amyloid-plaque load scoring system, and flutemetamol PET was classified as positive when one of the five brain regions (frontal, parietal, posterior cingulate/precuneus, striatum, and lateral temporal lobes) systematically reviewed for flutemetamol PET was positive in either hemisphere (Kim et al., 2018). To include participants with relatively pure pathology, we excluded 108 (18.3%) participants with Aβ- ADD, 75 (42.4%) participants with Aβ+ SVaD, and 88 (19.0%) participants with Aβ+ NC.

### Brain MRI scans

T2, T1, FLAIR, and T2^*^-weighted GRE MRI images were acquired from all participants at the SMC using the same 3.0-Tesla MRI scanner (Philips, Best, the Netherlands). An Achieva 3.0-Tesla MRI scanner (Philips) was used to acquire three-dimensional T1 turbo field echo MRI data from all study participants, with the following imaging parameters: sagittal slice thickness, 1.0 mm with 50% overlap; no gap; repetition time, 9.9 ms; echo time, 4.6 ms; flip angle, 8°; and matrix size, 240 × 240 pixels reconstructed to 480 × 480 over a field view of 240 mm.

### Neuropsychological tests

All participants underwent neuropsychological testing using the Seoul Neuropsychological Screening Battery 2nd edition for diagnostic purposes (Kang, and Na, 2012; Kang et al., 2019). The battery comprised tests for attention, language, calculation, praxis, visuospatial/constructive function, verbal/visual memory, and frontal executive function, as previously described (Seo et al., 2007).

### Measurement variables

Medical assessments were conducted by medically trained health professionals based on standard protocols. To evaluate the level of education, we inquired about participants' formal education in detail, including whether they had completed each educational level and the total duration of educational attainment. We identified the cardiometabolic risk factors as follows: hypertension (defined as the past medical history of hypertension or the participant taking any antihypertensive drugs at present), and diabetes mellitus (DM), defined as a previous history of DM or the participant currently taking insulin or oral anti-diabetic medications].

### Statistical analysis

The demographic and clinical differences between Aβ- NC and Aβ+ ADD, or between Aβ- NC and Aβ- SVaD, were analyzed using the Student's *t*-test and chi-square test. To investigate the relationship between the measurement variables and dementia status, we performed multivariable logistic regression analysis including age, sex, hypertension status, DM status, *APOE* ε4 carrier status, and education level as covariates.

Path analysis was performed using the Mplus software (version 8.0) to investigate whether vascular risk factors mediate the effect of education on the development of dementia. Since our model had binary dependent variables and mediators, we used the weighted least squares method, which is widely used in the analysis of categorical variables (Muthén and Muthén, 1998; MacKinnon et al., 2007). Indirect, direct, and total effects were calculated to examine the effect of vascular risk factors on the relationship between education level and the development of dementia. First, we fitted a saturated model with all associations. Subsequently, we considered years of education as the predictor, vascular risk factors as the mediators, and dementia as the binary outcome variable, after controlling for age and sex.

## Results

### Demographics and clinical characteristics

**Figure 1:**
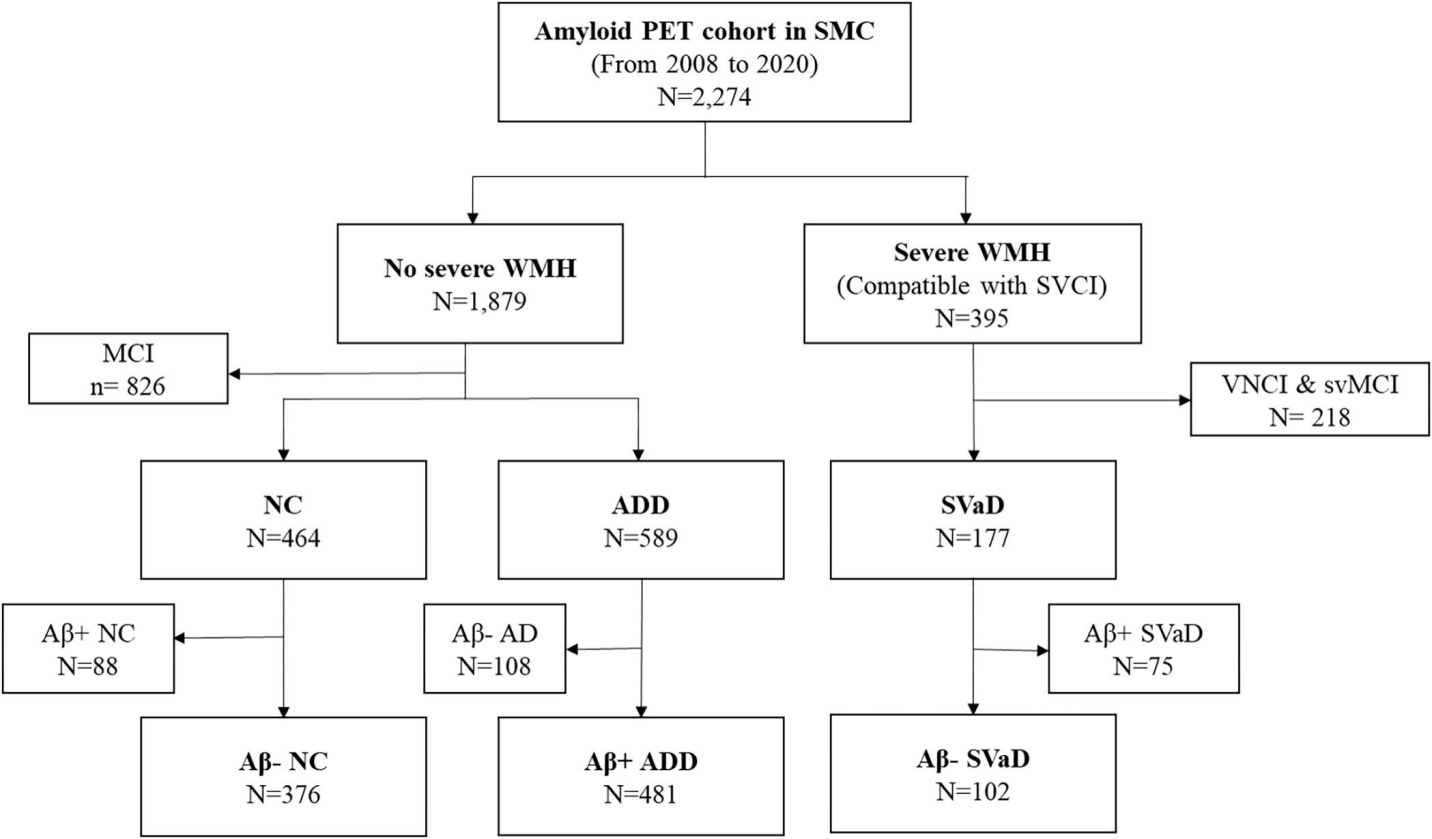
Flowchart for subjects selection. PET, positron emission tomography; SMC, Samsung medical center; WMH, white matter hyperintensities; MCI, mild cognitive impairment; VNCI, vascular no cognitive impairment; svMCI, subcortical vascular MCI; NC, normal cognition; ADD, Alzheimer's disease dementia; SVaD, subcortical vascular dementia.

The flowchart for the selection of study participants is shown in Figure 1. A total of 376 Aβ- NC, 481 Aβ+ ADD, and 102 Aβ- SVaD patients were finally included in the study. The clinical characteristics of the study participants are shown in Table 1. Aβ- SVaD (75.5 ± 7.1 years) was significantly older than Aβ- NC (70.1 ± 7.1 years) and Aβ+ ADD (70.0 ± 8.8 years) (*p* < 0.05). Aβ- SVaD had lower education (7.9 ± 5.1 years) than did Aβ- NC (11.8 ± 4.8 years) and Aβ+ ADD (11.2 ± 4.9 years) (*p* < 0.05). The prevalence of *APOE*4 carriers was higher in the Aβ+ ADD group (54.9%) than in the Aβ- SVaD (21.6%) and Aβ- NC (19.1%) groups. The frequencies of hypertension and DM were higher in the Aβ- SVaD group (78.4 and 32.4%, respectively) than in the Aβ+ ADD (41.8 and 15.8%, respectively) and Aβ- NC (44.4 and 20.8%, respectively) groups.

**Table 1:** Demographics and clinical characteristics.

**Characteristics at the initial visit**	**Aβ- NC (*n* = 376)**	**Aβ+ ADD (*n* = 481)**	**Aβ- SVaD (*n* = 102)**
Age, y	70.1 ± 7.1 (55–87)	70.0 ± 8.8 (55–97)	75.5 ± 7.1 (55–92)^a, b^
Sex, female	241 (64.1)	292 (60.7)	70 (68.6)
Education years	11.8 ± 4.8 (0–21)	11.2 ± 4.9 (0–21)	7.9 ± 5.1 (0–18)^a, b^
Hypertension	167 (44.4)	201 (41.8)	80 (78.4)^a, b^
Diabetes mellitus	77 (20.5)	76 (15.8)	33 (32.4)^a, b^
*APOE* ε4 carriers	72 (19.1)	264 (54.9)^a^	22 (21.6)^b^

^a^p < 0.05 compared to NC.

^b^p < 0.05 compared to ADD.

NC, normal cognition; ADD, Alzheimer's disease dementia; SVaD, subcortical vascular dementia.

Values are expressed as means ± standard deviations (range) or numbers (%).

### Relationships between education and dementia

Table 2 shows the results of multivariable logistic regression analysis for the clinical predictors of the Aβ+ ADD and Aβ- SVaD groups. An increased age independently predicted Aβ- SVaD [odds ratio (OR) 1.100, 95% confidence interval (CI), 1.059–1.143], but not Aβ+ ADD (OR 0.997, 95% CI, 0.979–1.015). An increase in the number of years of education was independently associated with Aβ- SVaD (OR: 0.866, 95% CI: 0.824–0.911) but not with Aβ+ ADD (OR 0.971, 95% CI 0.940–1.003). The presence of *APOE4* carriers predicted Aβ+ ADD (OR 5.158, 95% CI, 3.760–7.075), but not Aβ- SVaD (OR 1.046, 95% CI, 0.556–1.967). The frequency of hypertension was independently associated with Aβ- SVaD (OR 3.373, 95% CI, 1.908–5.961), but not with Aβ+ ADD (OR 0.884, 95% CI, 0.653–1.196).

**Table 2:** Odds ratios of risk factors for ADD and SVaD.

**Risk factor**	**Aβ- NC vs. Aβ+ ADD**	**Aβ- NC vs. Aβ- SVaD**
	**Odds ratio (95% CI)**	**Odds ratio (95% CI)**
Sex, female	0.702 (0.507–0.971)	0.879 (0.501–1.542)
Age, y	0.997 (0.979–1.015)	1.100 (1.059–1.143)
Education, y	0.971 (0.940–1.003)	0.866 (0.824–0.911)
Hypertension	0.884 (0.653–1.196)	3.373 (1.908–5.961)
Diabetes mellitus	0.677 (0.462–0.991)	1.628 (0.926–2.862)
*APOE* ε4 carriers	5.158 (3.760–7.075)	1.046 (0.556–1.967)

*NC, normal cognition; ADD, Alzheimer's disease dementia; SVaD, subcortical vascular dementia; CI, confidence interval*.

### Results of path analysis

**Figure 2:**
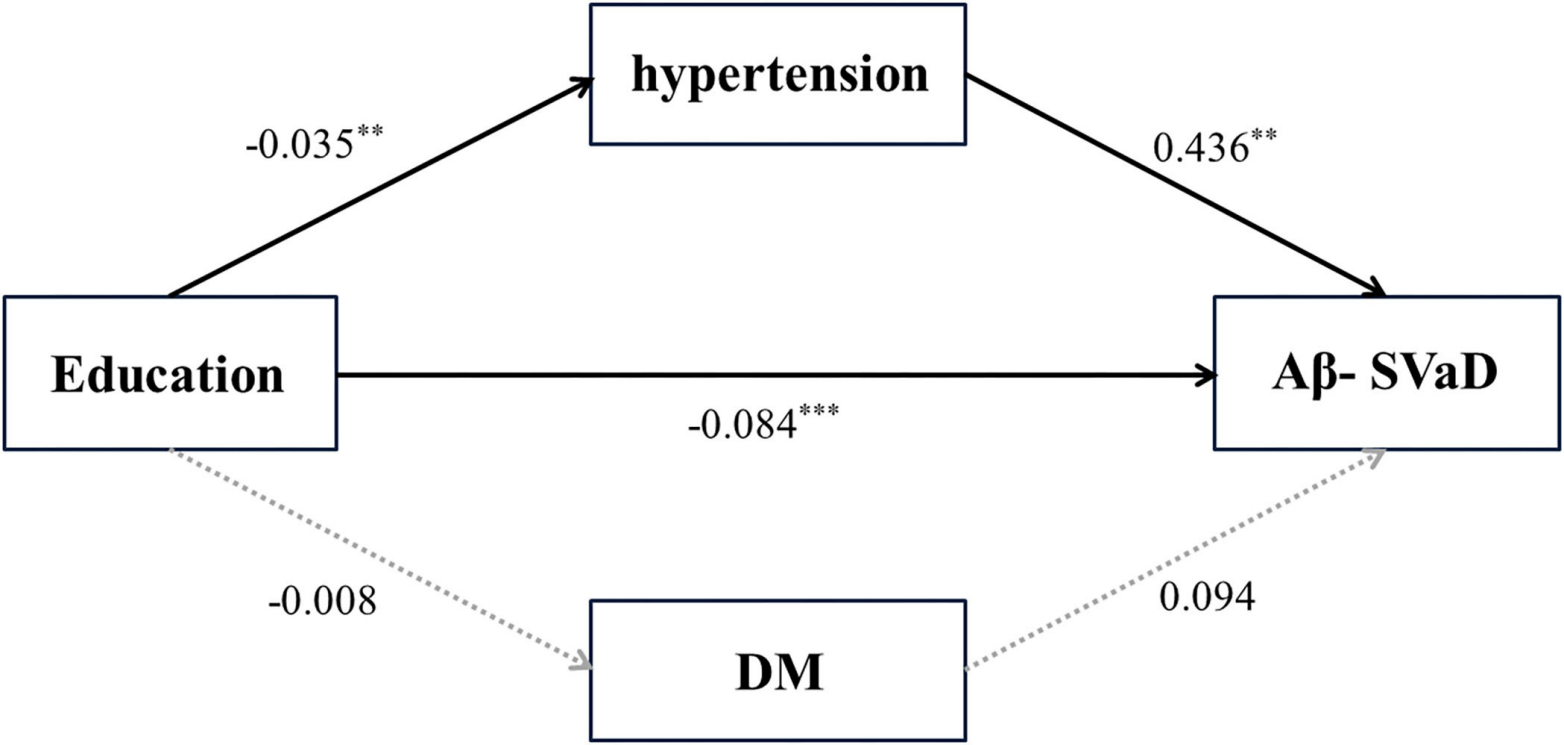
Path analysis of education levels and vascular risk factors for dementia. DM, diabetes mellitus, SVaD, subcortical vascular dementia; β, unstandardized beta; SE, standard error. **p-value < 0.005, ***p-value < 0.001.

To verify whether hypertension and DM act as mediators in the relationship between educational level and Aβ- SVaD, the dementia type which low education was only associated with, we analyzed a mediated model, as shown in Figure 1. In the relationship between education and Aβ- SVaD, lower education was associated with a higher risk of Aβ- SVaD [total effect, β = 0.090, standard error (SE)= 0.016, *p* < 0.001]. In a mediation analysis between education, hypertension, and Aβ- SVaD, lower education was associated with a higher risk of hypertension (*p* = 0.004), which was further associated with a higher risk of SVaD (*p* = 0.001) [indirect effect, β = −0.015; SE = 0.007; *p* = 0.037; Bootstrap 95% confidence interval (CI) −0.033, −0.004]. In addition, lower education was directly associated with a higher risk of SVaD, even without the mediation of hypertension (direct effect, β = −0.084; SE = 0.016; *p* < 0.001). In terms of the relationship between education, DM, and Aβ- SVaD, education was not associated with DM (*p* = 0.556), and DM was not associated with a risk of Aβ- SVaD (*p* = 0.480), which supported that DM did not mediate the relationship between education and Aβ- SVaD (indirect effect, β = −0.001; SE = 0.002; *p* = 0.759; Bootstrap 95% CI −0.007, 0.003). The chi-square test of model fit for the baseline model showed a value of 173.230 with *p*-value < 0.001 (Figure 2; Table 3).

**Table 3:** Relationship between education, vascular risk factors, and SVaD.

	**β**	**SE**	***p*-value**
**Education—SVaD**			
Direct effects			
Education → SVaD	−0.084	0.016	<0.001
**Education—Hypertension—SVaD**			
Direct effects			
Education → hypertension	−0.035	0.012	0.004
Hypertension → SVaD	0.436	0.137	0.001
Indirect effects			
Education → hypertension → SVaD	−0.015	0.007	0.037
**Education—DM—SVaD**			
Direct effects			
Education → DM	−0.008	0.013	0.556
DM → SVaD	0.094	0.133	0.480
Indirect effects			
Education → DM → SVaD	−0.001	0.002	0.759

Covariates: age, sex.

SVaD, subcortical vascular dementia; DM, diabetes mellitus; SE, standardized error.

## Discussion

In this study, we investigated the effects of vascular risk factors on the association between educational level and the risk of developing Aβ+ ADD or Aβ- SVaD. The major findings of our study are as follows. First, lower education levels were associated with a higher risk of Aβ- SVaD. Second, the presence of vascular risk factors, particularly hypertension, was also related to a higher risk of Aβ- SVaD, but not Aβ+ ADD. Finally, path analysis showed that the presence of hypertension, but not DM, partially mediated the relationship between education level and diagnosis of Aβ- SVaD. Considered together, our findings suggest that education level might influence the development of Aβ- SVaD through the brain-battering hypothesis, which particularly involves deleterious effects of hypertension. Furthermore, our findings suggest that suitable strategies, such as education and the prevention of hypertension, are needed to prevent Aβ- SVaD.

Our first major finding was that lower education level was associated with Aβ- SVaD, which is consistent with the results of previous studies. One previous study revealed that cerebral small vessel disease (CSVD), such as subcortical lacunar infarcts, white matter lesions, and macroscopic infarcts, was more frequent in individuals with lower education levels (64% incidence in individuals with a below high school education level vs. 37% incidence in individuals who had graduated high school) (Del Ser et al., 1999). These results suggest that lower education levels were correlated with increased risk for SVaD, as SVaD is characterized by extensive CSVD (Erkinjuntti et al., 2000). Another Aβ PET-based study also found that participants with Aβ- SVaD (8.1 years) have lower levels of education than did the participants with Aβ+ ADD (10.5 years) or Aβ- NC (13.9 years) (Yoon et al., 2013). Although lower educational attainment has been generally regarded as a risk factor for the clinical diagnosis of ADD, in this study, there was no significant correlation between lower levels of education and the diagnosis of Aβ+ ADD (Katzman, 1993; Munoz et al., 2000; Caamaño-Isorna et al., 2006; Sharp and Gatz, 2011). This contradicting result might be related to the different study populations, such as the inclusion of Aβ+ ADD in this study in comparison to a clinical diagnosis of ADD in previous studies (Alexander et al., 1997; Scarmeas et al., 2003; Kemppainen et al., 2008; Meng and D'Arcy, 2012). Our findings were also supported by previous studies revealing no significant association between lower education levels and amyloid pathology (Jansen et al., 2015; Gottesman et al., 2016; Insel et al., 2016; Landau et al., 2016). Therefore, we consider that high education is not a protective factor for AD pathology, alternatively, it might be due to selection bias which excludes low-educated individuals with AD pathology who do not visit a clinic.

In this study, as expected, we observed a positive association between hypertension and Aβ- SVaD. The mechanism and effects of hypertension on the risk of development of SVD and subsequent SVaD have been well-elaborated in previous studies (Caruso et al., 2019; Moretti and Caruso, 2020). One of the main pathomechanisms underlying SVD is arteriolosclerosis caused by hypertension and subsequent hypoperfusion. However, in terms of dementia progression in individuals with SVD, it is also argued that hypotension rather than hypertension is more important (Moretti et al., 2008; Caruso et al., 2019; Moretti and Caruso, 2020). In this study, we simply used the presence of hypertension as a predictor, instead of many hypertension-related risk factors such as pulse pressure, hypotension, or interaction with age, which should be investigated further to help in the interpretation of underlying mechanisms leading to SVaD. In terms of the relationship between hypertension and ADD, we did not find any relationship between hypertension and Aβ+ ADD, although previous studies have shown that hypertension increases the risk of the development of ADD (Kivipelto et al., 2002; Qiu et al., 2003; Skoog and Gustafson, 2006; Purnell et al., 2009). This inconsistency may have come from a lack of biomarker information in the previous ADD population. In fact, our findings were supported by previous studies that indicated that there was no significant association between hypertension and amyloid pathology (Gottesman et al., 2017). Unexpectedly, DM was not associated with the risk of Aβ- SVaD (Abner et al., 2015); rather, DM was negatively associated with Aβ+ ADD. Our findings might be related to previous studies showing a negative association between amyloid pathology and DM (Beeri et al., 2005; Nelson et al., 2009; Sonnen et al., 2009; Ahtiluoto et al., 2010; Kang et al., 2021). Therefore, it is possible that DM affects cognitive decline through non-amyloid pathology.

Our final major finding was that the presence of hypertension (but not DM) partially mediated the association between lower educational levels and the diagnosis of Aβ- SVaD. Our findings are consistent with the brain-battering hypothesis. That is, a lower level of education might be related to a lower socioeconomic status (SES), which might be related to higher exposure to toxins, and less healthy lifestyles, such as irregular or unhealthy diet, smoking and alcohol consumption, and poor access to medical care (Del Ser et al., 1999; Yoon et al., 2013). Furthermore, the development of hypertension and poorly-controlled hypertension induced by lower SES might eventually result in the development of Aβ- SVaD. Interestingly, another pathway was observed between lower levels of education and Aβ- SVaD, without the mediation of hypertension. This pathway might be related to the effects of lower educational attainment on the development of Aβ- SVaD through other unmeasured risk factors or imaging markers for detecting changes in brain structure and function, which we did not investigate. Additionally, the above mediation effects did not correspond to the relation between education, DM, and Aβ- SVaD. This might suggest that the presence of DM itself is not mainly affected by low education or related low SES, and it is not associated with Aβ- SVaD. We should look into the relation between DM and dementia more closely, by investigating whether unmeasured DM-related risk factors such as glycemic variability or hypoglycemia have a role in the development of different types of dementia.

The strength of our study lies in its well-characterized and large cohort of patients who underwent Aβ PET and structural MRI according to standardized protocols. However, our study had several limitations that should be addressed. First, because of the cross-sectional study design, the causal relationship between education level, vascular risk factors, and the development of ADD or SVaD remains unclear. Therefore, future longitudinal studies are required to determine temporal relationships. Second, because we intentionally excluded subjects with mixed dementia (Aβ+ SVaD) to clarify and separately investigate the relation between education, vascular risk factors, and either of two major pathologies, this study cannot be generalizable to mixed dementia cases which are common in old age. In contrast, we used Aβ PET and MRI for severe WMH to diagnose pure ADD and pure SVaD—the possibility of other mixed pathologies could not be ruled out, owing to the lack of histopathological confirmation. Third, we could not directly measure and consider SES in the model, which might partially explain the association between education and the risk of developing SVaD. Finally, we could not consider other newly-acknowledged vascular risk factors (such as homocysteine, folate, or vitamin deficiency) or the downstream imaging markers of brain pathological changes (such as the volume of WMH, number of lacunes, and cortical thickness), but instead used hypertension or DM as an independent variable and each specific dementia subtype (ADD and SVaD) as the dependent variable. Therefore, further research on path analysis, including these various variables as mediators, is needed to understand the association between education, vascular risk factors, and each pathological marker. However, our study is noteworthy because we included biomarker-guided diagnoses of ADD and SVaD and used a simple study design to demonstrate the association between education level, hypertension, and SVaD.

## Data availability statement

The original contributions presented in the study are included in the article/supplementary material, further inquiries can be directed to the corresponding author/s.

## Ethics statement

The studies involving human participants were reviewed and approved by Samsung Medical Center. The patients/participants provided their written informed consent to participate in this study.

## Author contributions

Conceptualization: SH, HJ, HK, DN, and SS. Methodology: SH, HJ, and SS. Formal analysis and investigation: SH, YK, YP, and HK. Writing—original draft preparation: SH and HJ. Writing—review and editing: SH, YK, HJ, and SS. Funding acquisition: SS. Supervision: HJ, DN, and SS. All authors contributed to manuscript revision, read, and approved the submitted version.

## Funding

This research was supported by a grant of the Korea Health Technology R&D Project through the Korea Health Industry Development Institute (KHIDI), funded by the Ministry of Health and Welfare and Ministry of Science and ICT, Republic of Korea (grant number: HU20C0111), the Ministry of Health Welfare, Republic of Korea (grant number: HR21C0885), and the National Research Foundation of Korea (NRF) grant funded by the Korea government (MSIT) (NRF-2020R1A2C1009778). This study was also supported by Future Medicine 2030 Project of the Samsung Medical Center (#SMX1210771), a grant of the Korean Health Technology R&D Project, Ministry of Health and Welfare, Republic of Korea (HI19C1132), and the National Institute of Health Research Project (2021-ER1006-01).

## Conflict of interest

The authors declare that the research was conducted in the absence of any commercial or financial relationships that could be construed as a potential conflict of interest.

## Publisher's note

All claims expressed in this article are solely those of the authors and do not necessarily represent those of their affiliated organizations, or those of the publisher, the editors and the reviewers. Any product that may be evaluated in this article, or claim that may be made by its manufacturer, is not guaranteed or endorsed by the publisher.
